# Supplementary material for: A qualitative investigation of young people’s experiences and views of Early Support Hubs across England
Source: PLoS One. 2026 May 29;21(5):e0347789. doi: 10.1371/journal.pone.0347789 (PMC13221038; doi:10.1371/journal.pone.0347789)
Supplement: S1 File — (DOCX) [file pone.0347789.s001.docx]

# S1 File: Topic guide for interviews with service users

*[Start recording]*

## Part 1: Use of early support hubs

1. What sort of difficulties led you to use [hub]?

Potential prompts:

- - Would you consider yourself to have any mental health difficulties?

1. When did you first and last access a support hub?

1. How often have you used [hub]?

Potential prompts:

- - Frequency of visits? Eg. once a week, one-off?
  - Number of visits overall?

## Part 2: Journey to accessing early support hubs

1. Could you tell me about your journey to receiving support from [hub] for the first time?

Potential prompts:

- - How did you find out about [hub]? Eg. recommend by school/friends/family?
  - Other support you had accessed/tried to access before [hub]?
  - Reasons for choosing [hub] over other services?

1. How easy or difficult was it to access support from [hub]?

Potential prompts:

- - Anything that made it easier?
  - Anything that made it harder?

## Part 3: Experience of using early support hub

1. Could you tell me about the kind of support you have received from [hub]?

Potential prompts:

- - Experiences of drop-in advice/therapy or counselling/groups?
  - Did it meet your expectations? Why/why not?

1. How well has the service worked for you?

Potential prompts:

- - Parts you particularly liked?
  - Parts you didn’t like so much?
  - How relevant/sufficient did support feel?
  - Any issues you wanted help with that hub didn’t provide support for?
  - How well did the location/physical environment work for you?
  - What sort of changes in mental health/wellbeing/daily life, if any, did you experience as a result?

1. What were experiences with staff like?

Potential prompts:

- - Any issues?

1. To what extent were your family involved in support at [hub]?

Potential prompts:

- - Any specific opportunities for family involvement at hub?
  - Thoughts/feelings about family being involved/not involved?

1. Reflecting on your overall experiences, would you recommend [hub] to other young people? Why/why not?

## Part 4: Future recommendations for early support hubs

1. How did your experience with [hub] compare to other types of support you have previously used or tried to use?

*(To be asked unless clear from Q4 that there has been no use/attempted use of any other services)*

Potential prompts:

- - Any ways hub was more helpful?
  - Any ways hub was less helpful?

1. In an ideal world, what would the perfect hub be like?

Potential prompts:

- - Process for accessing hub?
  - Types of support on offer?
  - How support would be delivered?
  - What would it physically look like (eg. waiting rooms, support rooms, location)?
  - Improvements to current hub that would be needed?

## Part 5: Closing questions

13. Is there anything else you would like to share about your experience of using an early support hub or your views about what works well?

*[End recording]*
