# Supplementary material for: A qualitative investigation of young people’s experiences and views of Early Support Hubs across England
Source: PLoS One. 2026 May 29;21(5):e0347789. doi: 10.1371/journal.pone.0347789 (PMC13221038; doi:10.1371/journal.pone.0347789)
Supplement: S2 File — (DOCX) [file pone.0347789.s002.docx]

**S2 File: De-identified coding framework**

| **Name** | **Description** |
| --- | --- |
| **ACCESSIBILITY** | Theme including conversation around what it was like to reach out to the hub and receive support. |
| Barriers to accessing support | Factors that made it more difficult to receive support from the hub. |
| Anxiety, fear or frustration | Include any mention of difficult emotions felt when reaching out for support from hub for the first time. |
| Attributing difficulties to factors external to the hub | Barriers to receiving support that were not at the level of the support hub itself, e.g. individual-level factors of anxiety, or broader barriers such as problems with the local council. |
| Having to physically go to the hub | Include any mention of needing to attend the hub in person, including practical, emotional, or logistical aspects of physically going to the space. |
| Lack of awareness and clarity about hub | Mention of young people not knowing about the hub or not having a clear idea of what the hub does. |
| Awareness, visibility and clarity on accessing the hub | Discussion around young people knowing or finding out about the hub. This includes mention of whether young people would be able to find out about the hub by walking past the building. |
| Clarity about what the hub does | Mention of wanting hub to be clearer regarding its service offerings e.g. making it more obvious that they offer drop-in support rather than long term therapy. |
| Transparency with young people accessing the hub | Include any mention of how clearly and openly information about the hub, its processes, and what to expect was communicated to young people. |
| Parental consent requirement | Include any mention of requirements for parental or guardian consent to access the hub, including how this was communicated or experienced. |
| Wanting to cope without external support | Include any mention of wanting to manage difficulties independently without seeking support from the hub or other services. |
| Youth violence | Include any mention of youth violence affecting access to or engagement with the hub. |
| Language barriers | Include any mention of language barriers affecting access to or engagement with the hub. |
| Easy to access | Mention of factors that made it easier to receive support from the hub. |
| Accessible location | Include any mention of the accessibility or convenience of the hub’s location. |
| Easing you in or getting easier over time | Include any mention of finding it easier to access or engage with the hub over time. |
| Easy referral process | Include any mention of the referral process being easy or straightforward. |
| Flexible usage, drop in use, ad hoc use | Mention of being able to drop into the hub as and when you need it rather than having to follow a strict routine of usage e.g. once a week. |
| Quick way to get support | Discussion around time it took between reaching out and actually receiving support. |
| Support is free | Discussion around not having to pay for support at hub. |
| Warm atmosphere and staff approach | Include any mention of the hub’s atmosphere or staff approach being warm, friendly, or welcoming. |
| How young people find out about the hub | Mention of how a young person found out about their hub e.g. from a friend, through online research, through school. |
| Preferred mode of delivery | Mention of how a young person prefers support to be delivered e.g. online, face-to-face. |
| BEYOND CLINCAL INTERVENTIONS – A HOLISTIC APPROACH TO WELLBEING | Theme including conversation around hub offering support for everything a YP may need rather than just mental health support, and being readily available for whenever a young person needs them. |
| Breadth of support | Mention of variety and range of support on offer from hub. |
| Connecting with other services | Mention of hub communicating with, or referring on to, other services when a young person comes with an issue that the hub cannot manage alone. |
| Wanting more collaboration between different services and professionals | Include any mention of wanting greater collaboration between different services or professionals. |
| First using hub for one thing but over time ending up using it for another | Include any mention of using the hub initially for one type of support or reason and later using it for different needs over time. |
| Wanting all different types of support in same space | Mention of wanting to be able to access various different types of support (e.g. mental health support, financial support, social support) within a single service. |
| Flexible usage, drop in use, ad hoc use | Mention of being able to drop into the hub as and when you need it rather than having to follow a strict routine of usage e.g. once a week. |
| Concerns about abusing the system | Mention of not wanting to overuse or take advantage of the flexible/drop-in hub model. |
| Individualised support and flexible ways of access to meet young people where they are | Include any mention of individualised support and flexible ways of accessing and engaging with the hub to meet young people’s needs. |
| Making the most of the hub | Mention of using the service as often as they can. |
| Holistic impact of hub support | Mention of any perceived consequences of the support received. |
| Changes to socioeconomic circumstance | Mention of tangible socioeconomic changes including securing employment, housing, funding. |
| Confidence | Mention of the impact of the hub or support on young people’s confidence. |
| Coping mechanisms | Mention of the hub helping young people develop coping mechanisms. |
| Creative space | Mention of hub helping young people explore creative or artistic interests and passions. |
| Desire to help others | Mention of hub inspiring young people to help other people facing similar circumstances. |
| General positive change to wellbeing | Include any mention of hub leading to general improvements in wellbeing, mental health, or daily life. |
| Having a fun time | Mention of positive, enjoyable, or fun experiences when accessing or using the hub. |
| Help when you're feeling lost | Mention of hub helping young people to find their way in life e.g. gaining stronger sense of self, knowing how to make desired life changes. |
| Helped problems with eating | Include any mention of the hub helping with difficulties related to eating. |
| Instilling hope | Mention of increased hopefulness about the future as a result of accessing or engaging with the hub. |
| Less anxious | Mention of feeling less anxious due to hub support. |
| Life skills, daily functioning and coping | Mention of developing life skills, daily functioning, or coping strategies through support from the hub. |
| Meeting friends or similar others | Mention of meeting friends or peers, including connecting with others with similar experiences through the hub. |
| Navigating sexuality | Include any mention of support with understanding or navigating sexuality. |
| Physical health | Mention of positive impact on physical health or physical wellbeing through engaging with the hub. |
| Reducing substance use | Include any mention of reductions in substance use. |
| Self-belief and self-worth | Mention of changes in self-belief, self-worth, or self-esteem associated with using the hub. |
| Setting boundaries | Mention of learning about, developing, or implementing boundaries in relationships or interactions. |
| Understanding others’ emotions | Mention of improved understanding of other people’s emotions or perspectives. |
| Initial reason for hub use | Include any mention of reasons for first accessing the hub. |
| Issues with other services | Mention of choosing hub due to difficulties experienced with different services they had used prior. |
| Mental health needs | Mention of first using hub for mental health needs. |
| General mental health support | Mention of first using hub for general mental health support. |
| Mental health crisis | Mention of first accessing hub during mental health crisis e.g. after calling 111. |
| Wanting therapy or counselling | Mention of first contacting hub in attempt to access psychological therapy. |
| Multiple needs | Mention of first using hub for multiple different reasons e.g. both mental health support and financial support. |
| Referred by somebody else | Mention of first accessing hub due to somebody else's recommendation or referral. |
| Socioeconomic difficulties | Mention of first using hub for support with social or financial difficulties e.g. homelessness, employment support, funding. |
| Trying something new | Mention of first using hub because young person wanted to try a form of support that they had never tried before. |
| Issues with more general informal approaches | Include any mention of difficulties or concerns with more informal approaches used in the hub. |
| Lack of staff qualifications | Mention of staff members' professional training or qualifications. |
| Limited specialisation | Mention of limited specialisation within the hub or among staff/provision. |
| Non-clinical environment (physical) | How the physical hub environment differs from traditional clinical or medical environments. |
| Non-clinical philosophy or approaches | How the general approach and frameworks used by hubs do not follow traditional medical or clinical models. |
| Feeling like an individual person, not a number or label | Mention of feeling valued as a unique individual, rather than simply as a mental health case or number on a list. Covers topics like individualised/personalised support too. |
| Having someone to talk to or someone to listen as main part of support | Mention of the value of having someone to talk to or to simply listen to you rather than offer a specific therapy or intervention. |
| Lack of staff qualifications | Mention of staff members' professional training or qualifications. |
| Less formal, professional and scripted support, more subtle and relaxed | Mention of hub staff communicating with young people in a more casual and friendly manner as opposed to in a more restricted and professional manner. E.g. in other services it feels like staff 'read off a sheet' whereas in hubs they 'just say what they think'. |
| Do not like formalities e.g. questionnaires | Include any mention of disliking formalities in the support process, such as questionnaires or structured forms. |
| Support outside of traditional therapy | Include any mention of the hub providing support that is outside of traditional therapy approaches. |
| Range of service offerings | Descriptive list of types of support young people mention their hub offering. |
| Abuse |  |
| Advice for parents wanting to support their children |  |
| Advocacy |  |
| Drop in |  |
| Education |  |
| Employment |  |
| Filling out applications and forms |  |
| Financial support |  |
| Food vouchers |  |
| Fun and games |  |
| Groups |  |
| Housing support |  |
| Information, advice, guidance (IAG) sessions |  |
| LGBTQ+ |  |
| Life skills |  |
| Loneliness or social connectedness |  |
| Mental health support |  |
| Opportunities to help others |  |
| Physical health |  |
| Political involvement |  |
| Relationships |  |
| Seeing individual staff members |  |
| Service transition support |  |
| Sexual health |  |
| Substance abuse |  |
| Volunteering |  |
| Young Ambassadors |  |
| Young parents |  |
| COMMUNITY, FRIENDSHIP AND CONSISTENCY | Discussion around young people feeling a sense of belonging, value and humanness from using the hub, and the consistency of the support provided. |
| Bonds and 'friendship' with staff e.g. ‘they know me’ | Mention of hub staff communicating with young people like they are a peer or a friend and treating them like they really know them as an individual. |
| Consistency, being there for you, checking in and caring | Include any mention of consistent support from the hub, including being available, checking in, or showing care. |
| Support offered whilst waiting for formal therapy | Mention of alternative support being offered (e.g. groups, drop-in service) whilst a young person is on the wait list for a specific intervention at the hub. |
| Hub becoming part of young person | Include any mention of the hub becoming part of a young person’s life, routine, or identity. |
| Hubs as safe, trustworthy, judgement free | Includes discussion around hubs providing a safe space where they can trust that staff won't judge them and that they will keep confidentiality when appropriate. |
| Meeting friends or like-minded people | Discussion around meeting other young people and forming friendships or feeling less alone. |
| Opportunities to help others | Mention of the hub providing chances to help other young people e.g. getting involved in Fund the Hub Campaigns, or co-leading support groups. |
| Referring friends or other young people to hub | Mention of recommending the hub to a friend who could benefit. |
| Staffing changes | Experiences of old staff leaving and new staff joining the hub. |
| YOUTH-CENTRED APPROACH | Theme including conversation around person-centred approaches where young people are is encouraged to make own choices about their support. |
| Achieving change independently, with hubs playing supporting role | Discussions around achieving a goal with the support of the hub but the hub not being the sole reason for that success, e.g. also recognising the hard work that they put in as an individual. |
| Family involvement | Mention of family involvement or any conversation about family members being involved in support received at hub. |
| Lack of pressure | Reference to not feeling forcefulness or pressure from the hub or staff members. |
| Listening and validating rather than telling or forcing | Mention of hub providing a space to vent and someone to listen, validate and affirm the young person’s experience and perspective, without staff then telling the young person what to do or enforcing a different perspective. |
| No pressure to talk to someone | Mention of being able to go and sit in the hub without a staff member forcing you to talk and open up to them. |
| Power dynamics with staff | Include any mention of perceived differences in power, authority, or control between staff and young people in the hub context. |
| Youth-led activities | Include any mention of activities led or organised by young people within the hub. |
| Young people involved in decision-making | Include any mention of young people being involved in decision-making within the hub. |
| Choice over support | Whether young people were given options regarding the type of support they would receive. |
| Decision making about hub | Mention of young people influencing decision-making about the hubs (e.g. activities, design, how it runs, staffing). |
| Hubs prefer self-referrals | Mention of self-referral being encouraged, supporting young people’s autonomy and decision-making about engagement with the hub. |
| Lack of choice over support | Mention of not being given options regarding the type of support they received, e.g. just being told that they will receive CBT. |
| HUBS AS LIMITED BY THEIR SCALE | Includes discussions around what aspects of support did not work so well, and any potential hub improvements. |
| The need to expand the hubs | Theme including mention of wanting to extend the support hub model, including expanding current hubs but also opening more hubs. |
| Increasing demands and worries about future | Mention of growing demands for youth mental health support in recent years and reflections on the consequences of this, e.g. worries about how hubs will continue to meet the needs of young people. |
| Increasing numbers who can access support | Conversation around how hubs could be improved to ensure more young people can access them. |
| Hubs for over 25s | Mention of what support might look like when a young person becomes too old to use the hub. |
| Hubs size and scale | Mention of the small size of existing hubs and a desire to expand them. |
| Need for more staff or not being able to be seen | Discussion around limited number of staff members which may mean young people cannot be seen when they want. |
| We need more hubs | Mention of wanting there to be more hubs. |
| Increasing support on offer | Discussion around wanting support with more issues, or more types of staff offering support etc. |
| Time constraints | Discussion around support being time limited e.g. limited number of counselling sessions, limited duration of sessions, limited opening hours. |
| Counselling support as time-limited | Discussion around time constraints of counselling sessions. |
| Harder to access regular support | Mention of difficulty scheduling frequent sessions at hub. |
| Limited drop-in opening hours | Mention of drop-in opening times being limited or wishing they were open at different times or on different days. |
| Waiting | Conversation around experience of waiting for support at the hub. |
| Drop-in wait times | Mention of waiting to see a staff member when they attend the drop-in service. |
| Want more things to do while waiting to be seen | Mention of wanting more activities to keep busy whilst waiting in drop in e.g. colouring books, sensory toys. |
| Long referral processes | Mention of waiting for a referral to go through that the hub has made to a different service. |
| Support offered whilst waiting for formal therapy | Mention of alternative support being offered (e.g. groups, drop-in service) whilst a young person is on the wait list for a specific intervention at the hub. |
| Waiting for specific therapy or counselling | Discussion around being on wait lists to receive a specific psychological intervention at the hub. |
| MISCELLANEOUS | Include any relevant content that does not clearly fit into other existing codes. |
| Can't fault them | Comments around not being able to think of any negatives about the hub. |
| Communication after appointment | Mention of wanting to receive a session summary from staff. |
| Comparisons with other services | Bucket theme including any comparisons made between hub and other services the young person has accessed |
| Better mode of delivery | Include any mention of the hub offering a preferred or more effective mode of delivery compared with other services. |
| Caters better for young people than adult services | Include any mention of the hub being better suited to young people’s needs compared with adult services. |
| Comparing time constraints | Include any mention of differences in time limits, availability, or flexibility compared with other services. |
| Less clinical | Include any mention of the hub being experienced as less clinical compared with other services. |
| Less dismissive or more validating | Include any mention of the hub being experienced as more validating or less dismissive than other services. |
| Less professional or formal, more humanising and personal | Include any mention of the hub being perceived as less formal or professional and more personal, relational, or humanising than other services. |
| More accessible | Include any mention of the hub being more accessible compared with other services. |
| More choice and autonomy rather than being told what to do | Include any mention of greater choice, autonomy, or collaborative decision-making compared with other services. |
| More holistic | Include any mention of the hub providing more holistic support compared with other services. |
| More inclusive | Include any mention of the hub being more inclusive compared with other services. |
| More knowledgeable | Include any mention of the hub staff being perceived as more knowledgeable compared with other services. |
| Most successful support to date | Include any mention of the hub being viewed as the most effective or successful support experience compared with previous services. |
| Safer, more confidential, more judgement free | Include any mention of the hub being experienced as safer, more confidential, or less judgemental compared with other services. |
| Similar to that offered by other services | Include any mention of the hub being similar to other services in terms of support or approach. |
| Expectations | Mention of the expectations of the hub held before they first accessed it |
| Didn't meet expectations | Mention of hub support not meeting their expectations. |
| Different from expectations | Mention of hub support and/or experiences being different to their expectations. |
| Lack of expectations | Mention of not knowing what to expect from the hub/not having any specific expectations. |
| Met expectations | Mention of hub support meeting their expectations. |
| Surpassed expectations | Mention of hub support exceeding their expectations. |
| Frequency and time frame for hub use | Mention of when a young person first and last used a hub, and how often they use it. |
| Frequency of use | Including reference to regular appointments. |
| Time frame for accessing hub | Mention of when the young person first and last accessed the hub. |
| Hub atmosphere | Including mention of the hub's non-physical environment, e.g. the 'feel' of the hub |
| Comfortable, welcoming, relaxing environment | Any mention of how the hub space feels comfortable, welcoming, or relaxing. |
| Environment itself as therapeutic | Mention of benefits felt just by virtue of being in the hub space, rather than by virtue of any specific staff support received. |
| Inclusive | Mention of hub feeling like a space that is accepting of all individuals e.g. with reference to race, gender, sexuality. |
| Loud, overwhelming | Include any mention of feeling overwhelmed by noise, activity, or sensory aspects of the hub environment. |
| Physical hub environment | Comments around physical features of hubs e.g. buildings, rooms, furniture, equipment. |
| Features and furnishings of the hub | Mention of any physical features of the hub e.g. art on walls, sofas, colour schemes in rooms. |
| Hub building and rooms | Discussion of the physical building at the site, including discussions of its accessibility, how open or closed off it feels etc. |
| Hub location | Any mention of the hub’s location and how well it suits young people. |
| Lack of privacy | Include any mention of privacy concerns or limited private space when accessing or receiving support at the hub. |
| Need for renovation | Include any mention of the need for renovation or physical improvements to the hub environment. |
| Nice, comfortable environment | Reference to how physical features of the hub create a comfortable setting. |
| Young people involved in design | Young people giving input regarding design and aesthetics of the hub. |
| Staff | Collection of codes around experiences with staff at the hub. |
| Giving feedback to staff | Include any mention of young people giving feedback to staff at the hub. |
| Importance of bonding with staff members | Mention of connecting with, bonding with, and trusting specific staff members. |
| Lack of training or qualifications | Mention of staff members' professional training or qualifications. |
| Staff backgrounds | Mention of personal background and experiences of staff e.g. religious background. |
| Staff roles | Mention of staff role titles e.g. mental health advisor, counsellor, receptionist. |
| Staff understanding, knowledge and skill | The knowledge base and understanding staff have of the topics young people want to address, and their ability to help young people deal with them. |
| Warm, welcoming, judgement free approach | Description of young person’s experience of the positive approaches taken by hub staff, including the approach feeling warm, welcoming, non-judgemental. |
| Types of mental health difficulties or neurodivergence | Disclosed mental health difficulties or neurodivergence. |
| What makes the ideal hub | Discussion of what would make an ideal hub. |
